# Simultaneous Material Segmentation and 3D Reconstruction in Industrial Scenarios

**DOI:** 10.3389/frobt.2020.00052

**Published:** 2020-05-22

**Authors:** Cheng Zhao, Li Sun, Rustam Stolkin

**Affiliations:** ^1^Extreme Robotics Lab, University of Birmingham, Birmingham, United Kingdom; ^2^Visual Computing Group, University of Sheffield, Sheffield, United Kingdom

**Keywords:** material segmentation, 3D material reconstruction, transfer learning, deep neural network, nuclear applications

## Abstract

Recognizing material categories is one of the core challenges in robotic nuclear waste decommissioning. All nuclear waste should be sorted and segregated according to its materials, and then different disposal post-process can be applied. In this paper, we propose a novel transfer learning approach to learn boundary-aware material segmentation from a meta-dataset and weakly annotated data. The proposed method is data-efficient, leveraging a publically available dataset for general computer vision tasks and coarsely labeled material recognition data, with only a limited number of fine pixel-wise annotations required. Importantly, our approach is integrated with a Simultaneous Localization and Mapping (SLAM) system to fuse the per-frame understanding delicately into a 3D global semantic map to facilitate robot manipulation in self-occluded object heaps or robot navigation in disaster zones. We evaluate the proposed method on the *Materials in Context* dataset over 23 categories and that our integrated system delivers quasi-real-time 3D semantic mapping with high-resolution images. The trained model is also verified in an industrial environment as part of the EU RoMaNs project, and promising qualitative results are presented. A video demo and the newly generated data can be found at the project website^1^ (Supplementary Material).

## 1. Introduction

Materials recognition is in high-demand in many industries, such as nuclear waste decommissioning and recycling in a circular economy. Take robotic nuclear waste decommissioning as an example. The legacy of nuclear waste clean-up is one of the largest environmental remediation problems in the UK as well as in Europe. An estimated over 100 billion pounds will be spent on waste clean-up over a few decades (of Commons Committee of Public Accounts, 2013). Humans can handle radioactive waste but only for limited periods and by wearing special air-fed protection suits, which then become contaminated. In other words, conventional nuclear waste decommissioning turns becomes an open-ended problem as more nuclear waste is generated. For these reasons, autonomous robotic nuclear waste sorting and segregation will be the only solution for reducing secondary waste.

Recognizing the material of which waste objects are composed is important in nuclear waste decommissioning, as different post-process and levels of segregation will be applied according to the material. For example, combustible materials (e.g., wood and clothing) can be burned, and deformable materials (e.g., rubber and plastic) can be melted and compressed. Our team is part of NCNR (the National Center for Nuclear Research) and works closely with the National Nuclear Lab on advanced robot perception and manipulation for waste decommissioning. This paper uniquely tackles the material recognition problem for the nuclear industry, and we propose a visual-based semantic segmentation approach to identify waste material categories in cluttered scenes.

Deep learning-based semantic understanding is the state-of-the-art in fundamental computer vision challenges, and large-scale annotation is required to learn a robust model to deal with the variability of the real world. However, in novel robotic applications, e.g., nuclear waste material recognition, very limited data can be provided because confidential nuclear data is not publicly available. Hence, leveraging public datasets and transferring the knowledge from other vision tasks to this novel application is highly desirable. Moreover, the capability to perform boundary-aware annotation and 3D semantic reconstruction can provide high-level semantic knowledge to robots, which allows the manipulator to dexterously fetch or remove waste objects from highly self-occluded heap or bin.

Facing these challenges, in this paper, we mainly focus on the following two problems. (i) Recognizing the material categories pixel-wise and simultaneously fusing per-frame recognition into a dense 3D map for robotic applications in the nuclear industry. (ii) Transferring knowledge from meta computer vision data to the material recognition problem and transferring knowledge from a relatively simple task (i.e., material categorization) to a more challenging task (i.e., boundary-aware material segmentation).

Specifically, we present a material semantic reconstruction system that can perform real-time 3D reconstruction while simultaneously recognizing and labeling each voxel according to its material in the generated dense 3D map. We evaluate the proposed approach using both a public material dataset and real-world industrial data from qualitative, quantitative, and running-time perspectives to verify the feasibility of the proposed system. The main contributions of this paper can be summarized as follows:

1. To the best of our knowledge, this is the first system to achieve simultaneous material recognition and dense scene reconstruction. It can integrate high-level semantic knowledge into conventional 3D geometry reconstruction.
2. The pixel-wise material segmentation is achieved via transfer learning from general object recognition to specific material recognition and from an image-wise classification task to a pixel-wise segmentation task. The proposed approach is end-to-end learned, without requirements for hand-crafted features or post-processing optimization.
3. The running-time performance of the well-implemented system can be boosted to around 10 Hz using a standard GPU, which is enough to deploy quasi-real-time material semantic reconstruction in industrial scenarios.
4. Because the large-scale material dataset, i.e., Materials in Context (MINC) (Bell et al., 2015), only provided very coarse annotated data for the material classification and segmentation, we generated high-quality new data including RGB image patches (821,092 patches for training, and 96,747 patches for testing) and fully pixel-wise annotated RGB images (1,498 images for training, and 300 images for testing). Those data are released as an important supplement of the MINC dataset for benchmarking material classification and segmentation research.

## 2. Related Work

Vision-based material understanding, including classification, segmentation, and reconstruction, has as yet been little investigated, even though it is highly desirable for industrial robotics applications, e.g., nuclear robotics. As it must deal with the variation in brightness and illumination in the real world as well as learning a generalizable model from observations, material recognition in unconstrained environments is known to be a challenging research task.

CURet (Dana et al., 1999) was the first material dataset to be established. This consists of 61 material categories, and each category is captured with images taken under 205 different illumination and pose conditions. Eric et al. proposed the KTH-TIPS (Hayman et al., 2004) and KTH-TIPS (Caputo et al., 2005) datasets as supplementary to CURet, providing variations in scale in addition to in pose and illumination. The Flickr Material Database (Sharan et al., 2009) provides 10 different material categories, with 100 different samples for each category. The GeoMat (DeGol et al., 2016) dataset is the first dataset to provide material images with 3D geometry data. However, all of the above datasets are built for material classification rather than the pixel-wise material segmentation. The Materials in Context (MINC) (Bell et al., 2015) dataset is the first large-scale material dataset that is of good diversity and is well-sampled across 23 category materials. It provides two kinds of annotated data: RGB image patches and pixel-wise labeled RGB images. Moreover, a 4D light field material dataset proposed by Wang et al. (2016) captures the material images from multiple viewpoints through a light-field camera.

For material classification, most previous research has employed hand-crafted visual features, e.g., reflectance-based edge features (Liu et al., 2010), pairwise local binary patterns (Qi et al., 2014), local binary patterns (Li and Fritz, 2012), and variances in oriented gradients (Hu et al., 2011) for classifiers such as Fisher Vector (FV) (DeGol et al., 2016) and Support Vector Machines (SVMs) (Hayman et al., 2004; Caputo et al., 2005). Since deep learning dominates the computer vision community, deep-learned features (Schwartz and Nishino, 2013; Cimpoi et al., 2014) are also adopted to achieve state-of-the-art accuracy of material classification. Moreover, DeGol et al. (2016) not only employ 2D visual features, e.g., texture and color, but also 3D geometrical features, e.g., surface normals, to improve the material classification. However, this research can only perform material classification with RGB patches, and pixel-wise material recognition, i.e., semantic segmentation of materials, is not achieved.

In order to achieve pixel-wise material recognition, Bell et al. (2015) converted CNN classifiers trained on image patches into an efficient fully convolutional framework with a fully connected conditional random field (CRF) for the material segmentation. Schwartz and Nishino (2016) took advantage of the abilities of both CNN and RNN to perform superior segmentation using local appearance and separately recognized global contextual cues, e.g., objects and places. Cimpoi et al. (2015) proposed a new texture descriptor, FV-CNN, obtained by Fisher Vector pooling of a CNN filter bank and achieved state-of-the-art performance on the Flickr Material Database (Sharan et al., 2009). Wang et al. (2016) utilized proposed new 4D light-field images to train an FCN with post-processing to achieve material segmentation. Further research (Giben et al., 2015; Purri et al., 2019) achieved interesting material segmentation applications on the Satellite and Railway Track images, respectively.

In contrast to our proposed approach, all of the above studies focus on material classification or segmentation without reconstruction so that the semantic information of material cannot be integrated into the 3D geometry map. The proposed approach in this paper can perform material segmentation and reconstruction simultaneously to generate a 3D semantic map. With the assistance of this high-level semantic (material) knowledge, a robot can perform robot-environment interactive tasks or motion planning in industrial scenarios.

## 3. Methodology

### 3.1. System Overview

**Figure 1:**
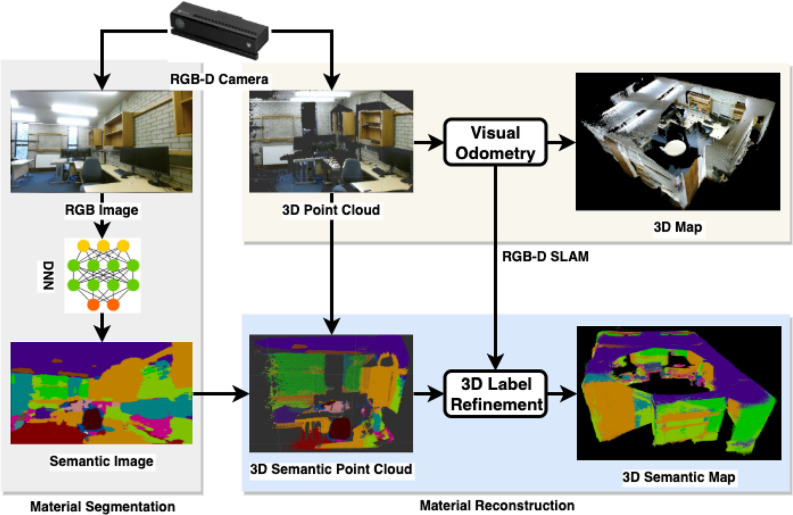
The pipeline of the proposed system of simultaneous material segmentation and reconstruction.

**Figure 2:**
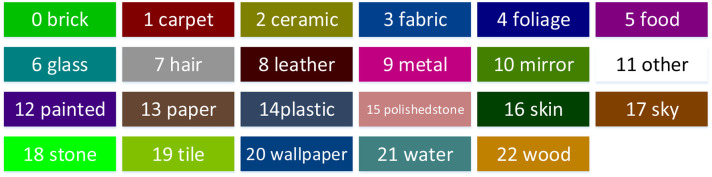
The color palette used in this paper.

This paper proposes a fully integrated system for material segmentation and reconstruction. It can perform real-time 3D dense mapping while simultaneously recognizing and labeling each point cloud in the map according to its material category. As Figure 1 shows, the system consists of two main parts: single-frame material segmentation and 3D semantic reconstruction (mapping). To be specific, the RGB image captured by the RGB-D camera is fed into a Deep Neural Network (DNN) to achieve pixel-level material segmentation. The semantic point cloud is then generated using the data pair of the semantically labeled RGB image and the corresponding depth image via back-projection. A sequence of semantic point clouds are incrementally combined via visual odometry, and meanwhile, the label probability of each point is refined by Bayesian updating. Finally, a dense 3D semantic map indicating voxel-wise material categories is generated. Please note that the color palette used in all of the Figures in this paper can be found in Figure 2.

### 3.2. Dataset and Data Preprocessing

The Materials in Context (MINC) dataset (Bell et al., 2015) is used to train and evaluate the proposed neural network. MINC is diverse and well-sampled across 23 categories, including ceramic, fabric, leather, stone, wood, etc.

Nuclear waste can be categorized into fuel waste and technical waste, and both are radioactive. The technical waste makes up more than 97% of the total waste and includes all types of waste produced during power generation, for example, liquid containers (such as bottles, cans), disposable protective items (e.g., suits, masks, gloves), and even construction materials (e.g., bricks and wood) used in the nuclear power station. Because nuclear waste containers are very expensive and space in a container is limited, the waste will be processed according to its materials to best utilize the space in containers. For example, wood and clothes can be burned, and the ashes can be stored, while objects like plastic bottles and metal cans can be compressed into blocks with small volumes. Therefore, material recognition is a critical task for nuclear waste sorting and segregation.

This paper focuses on recognizing the material categories of nuclear technical waste and the challenges of dealing with the variation of materials (i.e., inter-class dissimilarities) and the variability of the real world (e.g., brightness and illumination). In addition, we cannot obtain real nuclear technical waste at the current stage for network training. For these reasons, we choose to use a large-scale material dataset, MINC, which includes most kinds of materials found in technical waste, to train and evaluate our model.

**Figure 3:**
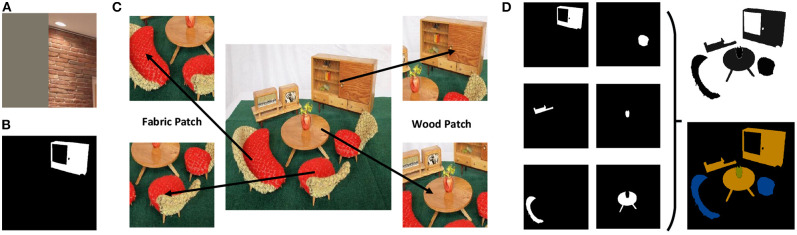
Data preprocessing. **(A)** A patch with NaN values in MINC. **(B)** A partially pixel-wise labeled image in MINC. **(C)** Extracting new patches from original images in MINC. **(D)** Combining the partially pixel-wise labeled images to generate a fully pixel-wise labeled image.

MINC provides two different types of annotations for training: a set of RGB patches with class labels, as shown in Figure 3A, and another set of partially pixel-wise labeled RGB images, as shown in Figure 3B. However, neither of these can be used directly for training end-to-end semantic segmentation network. There are many NaN values (shown as gray parts in Figure 3) in the original RGB patches, which give rise to a need for strong error supervision to prevent the classification network from converging. On the other hand, in the partially pixel-wise labeled RGB image, only the foreground object is labeled, whereas the background objects are masked. Thus, these images cannot provide contextual information during the training of the segmentation network.

Therefore, data preprocessing is applied to the MINC dataset. We first resize the original RGB image (500 × 500) and then extract RGB patches of different sizes (56 × 56, 156 × 156, 256 × 256, and 356 × 356) from it. This ensures that there are no NaN values in extracted patches and that only one type of material is at the center of each patch. Finally, 821,092 patches with corresponding class labels are generated for training, and 96,747 patches with class labels are generated for testing.

Next, we combine all of the partially pixel-wise labeled images that belong to the same original image to generate a single fully pixel-wise labeled image, as shown in Figure 3D. Since not all pixels are labeled in original images, the newly generated ground truth images are not 100% labeled. We further label all the unlabeled pixels, and repeated labeled pixels are treated as a category to be ignored during the training process. Finally, we generate 1,498 pixel-wise labeled training images and 300 pixel-wise labeled testing images. The size of the pixel-wise labeled ground truth images is also set to 500 × 500.

### 3.3. Material Classification

We first train a deep classification network using the generated RGB patches with the corresponding class labels. The VGG-16 (Simonyan and Zisserman, 2014) network, consisting of five convolution stacks and three dense connect layers, is employed for the classification task. However, the VGG-16 network is designed for the ImageNet challenge^2^ and thus can classify images into 1,000 object categories. We therefore modify the number of output nodes (i.e., the last dense connected layer of the VGG-16 network) to 23 instead of 1,000 for MINC material classification. Moreover, in order to accelerate the training, we transplant the weights of VGG-16 from the off-the-shelf pre-trained model^3^ on ImageNet to our neural network.

We provide performance evaluation of the classification using different sizes of patches in the experiment section. For feature representation learning, small patches can provide more texture information, while fully annotated images can provide more contextual information. Thus, the choice of patch size for the classification task is a trade-off between textural and contextual information.

### 3.4. Material Segmentation

**Figure 4:**
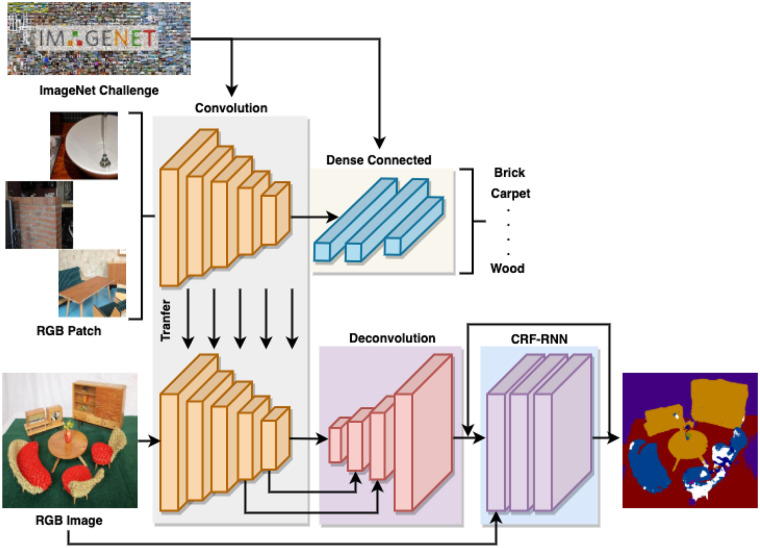
The architecture of the proposed network and transfer learning.

Next, we train a segmentation network using the generated pixel-wise labeled RGB images. As Figure 4 shows, the segmentation network consists of two sequential sub-networks: a Fully Convolutional Network (FCN) (Long et al., 2015) and Conditional Random Fields as Recurrent Neural Networks (CRF-RNN) (Zheng et al., 2015). FCN can provide a coarse semantic segmentation with prediction potentials to CRF-RNN, while CRF-RNN can smooth the label assignments between neighboring pixels to refine the coarse segmentation generated by FCN. Unlike the conventional approaches, which employ CRF as post-processing, we plugged in CRF-RNN after FCN as a unified framework that can be trained in an end-to-end way.

#### 3.4.1. FCN

FCN is a widely used end-to-end and pixel-to-pixel semantic segmentation network that consists of a convolution stack, a deconvolution stack, and a skip architecture. The convolution stack has the same architecture as the VGG-16 network truncated after pooling five layers. It can learn high-level semantic features with context cues by enlarging the receptive fields. However, it cannot retain significant boundary information on objects and structures due to the application of a series of pooling layers. The deconvolution stack can transform a feature map of the same size as the input RGB image. The skip architecture combines high-level and coarse semantic features from deep layers with low-level and fine features from shallow layers. Therefore, FCN can improve the performance of semantic segmentation by fusing the feature maps from both deep and shallow layers. However, FCN does not incorporate smoothness constraints between neighboring pixels so that it can only give a coarse pixel-wise prediction with some blob-like shapes.

#### 3.4.2. CRF-RNN

CRF-RNN means Conditional Random Fields as Recurrent Neural Networks, which is a hybrid model combining the learning property of CNN with the probabilistic graphical property of CRF. It can be inserted after a deep neural network to refine the coarse segmentation results generated.

Fully connected CRF (Krähenbühl and Koltun, 2011) takes account of contextual cues by minimizing the energy *E*(*x*) function to generate the most likely label assignment *x*. There are unary energy components and pairwise energy components in the Energy function *E*(*x*):

(1) $$\begin{array}{r} E\left(x\right)=\underset{i}{\sum }\psi_{u}\left(x_{i}\right)+\underset{i<j}{\sum }\psi_{p}\left(x_{i},x_{j}\right). \end{array}$$

The unary term ψ_*u*_(*x*_*i*_) obtained from the FCN measures the inverse likelihood of each pixel *i* assigning the corresponding label. However, the predicted pixel labels do not consider the smoothness or consistency of label assignments between neighboring pixels *j*. In contrast, the pairwise term ψ_*p*_(*x*_*i*_, *x*_*j*_) can penalize similar pixels that have different labels and encourage similar labeling of pixels with similar properties.

Pairwise potentials can be modeled as a linear combination of *M* Gaussian edge potential kernels $k_{G}^{\left(m\right)}$ using different weights ω^(*m*)^:

(2) $$\begin{array}{r} \psi_{p}\left(x_{i},x_{j}\right)=\mu \left(x_{i},x_{j}\right)\overset{M}{\underset{m=1}{\sum }}\omega^{\left(m\right)}k_{G}^{\left(m\right)}\left(f_{i},f_{j}\right). \end{array}$$

The Gaussian kernel $k_{G}^{\left(m\right)}$ is applied to feature vectors *f*_*i*_ of pixel *i*, e.g., spatial or color information. The label compatibility function is described as a Potts model μ(*x*_*i*_, *x*_*j*_) = [*x*_*i*_≠*x*_*j*_]. The Gaussian kernel $k_{G}^{\left(m\right)}$ in the pairwise potentials consists of a bilateral appearance potential and a spatial smoothing potential (*M* = 2):

(3) $$\begin{array}{r} k{\left(f_{i},f_{j}\right)}_{G}=\omega^{\left(1\right)}exp\left(-\frac{|p_{i}-p_{j}{|}^{2}}{2\theta_{\alpha }^{2}}-\frac{|I_{i}-I_{j}{|}^{2}}{2\theta_{\beta }^{2}}\right)+\omega^{\left(2\right)}exp\left(-\frac{|p_{i}-p_{j}{|}^{2}}{2\theta_{\gamma }^{2}}\right), \end{array}$$

where *p*_*i*_ and *p*_*j*_ refer to the spatial feature *x, y, z* and *I*_*i*_ and *I*_*j*_ refer to the color feature *R, G, B*. The parameters of Gaussian kernels are described using θ_α_, θ_β_, and θ_γ_.

Due to the consideration of pairwise potentials over all pixel-pairs in the whole image, minimizing the energy function in the fully connected CRF exactly is intractable. Therefore, the mean-field approximation is adopted to approximate the maximum posterior marginal inference. In CRF-RNN, one mean-field iteration can be formulated as a stack of common neural layers. The Initialization, Message Passing, Weighting Filter Outputs, Compatibility Transform, Adding Unary Potentials, and Normalizing operations in the mean-field iteration are implemented through Softmax, Convolutional, Convolutional, Convolutional, Concatenated, and Softmax layers, respectively. The repeated multiple mean-field iterations can be further formulated as a Recurrent Neural Network via repeating the above stack of layers.

In this work, the CRF-RNN is plugged in after the FCN to form a unified framework, and it is trained in an end-to-end manner. During the training process, the error differentials of CRF-RNN are passed to FCN via backward propagation through time, so that the FCN is able to generate better unary potentials for CRF-RNN optimization via forward propagation. More importantly, the parameters in CRF, e.g., the weights of Gaussian kernels and the label compatibility function, are automatically optimized during the full network end-to-end training.

### 3.5. Transfer Learning

The public VGG-16 model is well-trained using the large-scale ImageNet dataset and can classify objects from daily life belonging to 1,000 different categories. The learned knowledge from object classification should be helpful for the material classification. On the other hand, there are a huge number of sparsely labeled RGB patches (821,092) but a limited number of pixel-wise labeled RGB images (1,498) generated from the MINC dataset. Hence, we transfer the learned knowledge of the classification network to enhance the performance of the segmentation network via transfer learning.

As shown in Figure 4, there are two steps of knowledge transfer during the overall training process. The first step transfers the learned weights of the VGG-16 network pre-trained on ImageNet to the material classification network. The second step transfers the learned weights of the classification network, i.e., the VGG-16 network truncated after pooling five layers, to the segmentation network, i.e., the convolution stack of FCN. Both of them are implemented by learned network weights initialization followed by network fine-tuning. The first transfer learning focuses on the same network architecture but transfers the learned knowledge from object classification to material classification, while the second transfer learning focuses on two different network architectures but transfers the learned knowledge from a classification network to a segmentation network.

### 3.6. Material Reconstruction

A graph-based SLAM, i.e., RGB-D SLAM (Endres et al., 2014), is employed to achieve dense 3D material reconstruction. Given a semantic labeled image with the corresponding depth image, a 3D semantic point cloud (*X, Y, Z*) can be generated through back projection:

(4) $$\begin{array}{r} d_{u,v}\;\left[\begin{array}{c} u\\ v\\ 1 \end{array}\right]=\left[\begin{array}{c} f_{x}sc_{x}\\ 0f_{y}c_{y}\\ 0\;0\;1 \end{array}\right]\;\left[\begin{array}{c} X\\ Y\\ Z \end{array}\right], \end{array}$$

where (*u, v*) refer to the pixel position in the image plane and *d*_*u, v*_ refer to the corresponding depth value. *f*_*x*_, *f*_*y*_ refer to the focal length, and (*c*_*x*_, *c*_*y*_) refer to the principal point offset. *s* refers to the axis skew.

The visual odometry of RGB-D SLAM can estimate the ego-motion between two adjacent semantic point clouds and further enable an incremental semantic label fusion. Finally, using the global trajectory provided by the visual odometry, all of the semantic point clouds are combined together to generate a global semantic map. A Bayesian update is used for label hypothesis fusion using the multi-view semantic point clouds. Each voxel in a semantic point cloud stores the predicted label with the corresponding discrete probability. The voxel's label probability distribution is updated by means of a recursive Bayesian update:

(5) $$\begin{array}{r} P\left(x=l_{i}|I_{1,\ldots ,k}\right)=\frac{1}{Z}P\left(x=l_{i}|I_{1,\ldots ,k-1}\right)P\left(x=l_{i}|I_{k}\right), \end{array}$$

where *l*_*i*_ refers to the predicted label, *I*_*k*_ refer to the *k*th image, and *Z* refers to the constant for distribution normalization.

## 4. Experiments

In this section, the details of the network training process are first introduced. We then present performance evaluations of three different experiments: material classification, material segmentation on the MINC dataset, and material semantic reconstruction in an industrial scenario.

### 4.1. Network Training

We first train the VGG-16-based classification network using the newly generated 821,092 RGB patches of four different sizes, 56 × 56, 156 × 156, 256 × 256, and 356 × 356. The network weights are initialized using the public VGG-16 model pre-trained on ImageNet. Secondly, we train the FCN-32s, FCN-16s, and FCN-8s segmentation networks step by step using the newly generated 1,498 pixel-wise labeled 500 × 500 RGB images. The weights of the convolution stack in FCN are inherited from the fine-tuned VGG-16 model truncated after pooling five layers.

Finally, we insert the CRF-RNN after FCN as the bottom part of the whole network. After inheriting the learned FCN weights, the FCN with CRF-RNN network is trained again using the pixel-wise labeled RGB images in an end-to-end manner. During the training process, we set the number of mean-field iterations *T* to 5 in the CRF-RNN. This can reduce the training time and mitigate the vanishing gradient problem. During the test process, we set the number of mean-field iterations *T* to 5 or increase it to 10 according to the run-time required.

The parameters of all the trained networks, i.e., the learning rate, momentum, batch size, weight decay, and the type of training data, can be found in Table 1.

**Table 1:** The parameters of network training.

	**Learning rate**	**Momentum**	**Batch size**	**Weight decay**	**Training data**
VGG-16	1e-4 reduction with 0.1	0.95	50	0.0005	256 × 256 RGB patch
FCN-32s	1e-10	0.99	1	0.0005	500 × 500 RGB image
FCN-16s	1e-12	0.99	1	0.0005	500 × 500 RGB image
FCN-8s	1e-14	0.99	1	0.0005	500 × 500 RGB image
FCN with CRF-RNN	1e-12	0.99	1	0.0005	500 × 500 RGB image

### 4.2. Material Classification

The newly generated 96,747 RGB patches are employed for the material classification evaluation. We present the experimental results for the VGG-16 network trained by four differently sized patches in Table 2. It can be seen that the accuracy of classification initially increases but then decreases with increasing patch size. The optimal accuracy is reached when the patch size accounts for about 30–50% of the original image. The reason for the accuracy increasing initially is that more contextual cues become available with growth in the patch size, while the reason for the accuracy then decreasing is that there is a loss in spatial resolution with the growth of the path size. The best trade-off patch size for balancing the spatial resolution and contextual information is between 156 × 156 and 256 × 256 for the 500 × 500 images.

**Table 2:** The accuracy of material classification vs. patch size.

Patch size	56 × 56	156 × 156	256 × 256	356 × 356
Accuracy	69.20%	81.06%	80.18%	73.40%

*The best performance is in bold*.

### 4.3. Material Segmentation

The newly generated 300 pixel-wise labeled images are employed for the material segmentation evaluation. Following (Long et al., 2015), the standard parameters for semantic segmentation evaluation, namely pixel accuracy, mean accuracy, mean intersection over union (IoU), and frequency weighed intersection over union (IoU), are adopted for performance analysis. These metrics are defined as:

- Pixel accuracy: $\underset{i}{\sum }n_{ii}/\underset{i}{\sum }t_{i}$,
- Mean accuracy: $\left(1/n_{cl}\right)\underset{i}{\sum }n_{ii}/t_{i}$,
- Mean IoU: $\left(1/n_{cl}\right)\underset{i}{\sum }n_{ii}/\left(t_{i}+\underset{j}{\sum }n_{ji}-n_{ii}\right)$,
- Frequency weighted IoU: ${\left(\underset{k}{\sum }t_{k}\right)}^{-1}\underset{i}{\sum }t_{i}n_{ii}/\left(t_{i}+\underset{j}{\sum }n_{ji}-n_{ii}\right)$.

Here, *n*_*cl*_ refers to the number of classes, *n*_*ij*_ refers to the number of pixels of class *i* classified as class *j*, and $t_{i}=\underset{j}{\sum }n_{ij}$ refers to the total number of pixels belonging to class *i*.

#### 4.3.1. Qualitative Analysis

**Figure 5:**
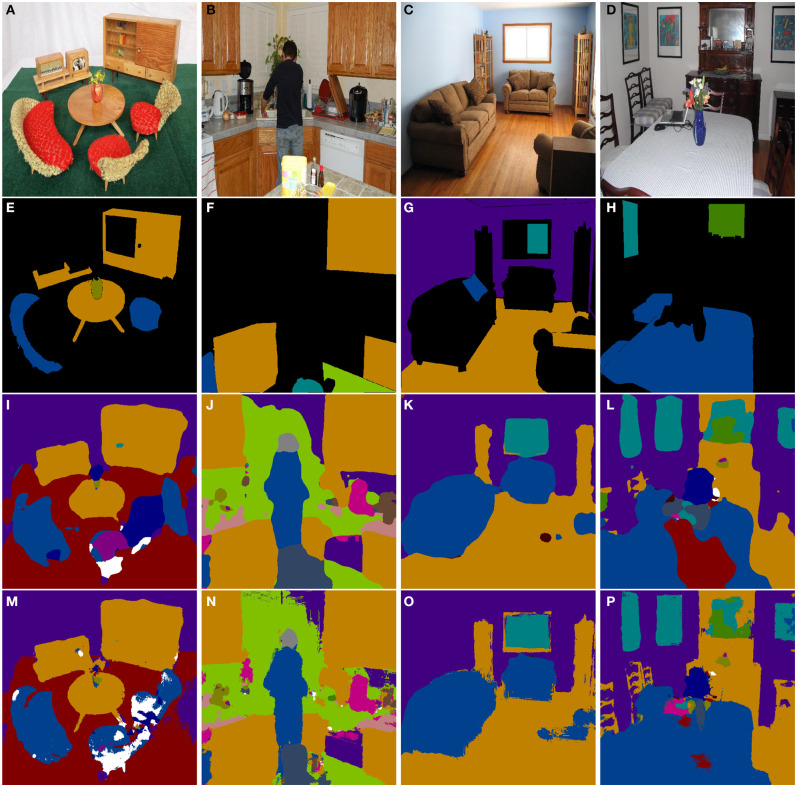
Material segmentation in MINC dataset. From left to right, top to bottom, the IDs of the sub-figures are **(A–P)**. The first row, i.e., **(A–D)**, are original RGB images, the second row, i.e., **(E–H)**, are ground truth images, the third row, i.e., **(I–L)**, are semantic segmentation results of FCN, and the fourth row, i.e., **(M–P)**, are semantic segmentation results of FCN with CRF-RNN.

The qualitative results of material segmentation on the MINC dataset are given in Figure 5. Due to the lack of neighborhood consistency constraints, there are a lot of non-sharp boundaries in the segmentation results of FCN. After plugging in CRF-RNN after FCN for the label assignment smoothing, the boundaries of the segmentation results are much clear compared with when using only FCN.

The first and second rows in Figure 5 show the original and ground-truth images on MINC. The third and fourth rows in Figure 5 show the 2D semantic segmentation results of FCN and of FCN with CRF-RNN, respectively. It can be seen that FCN with CRF-RNN generates semantic results with much clearer shapes than FCN alone, e.g., table legs in (Figure 5M), a person in (Figure 5N), a sofa in (Figure 5O), and a chair back and vase in (Figure 5P). In (Figure 5L), a large section of “fabric” is erroneously labeled as “carpet,” while the size of this erroneous area greatly decreases in (P) because of the neighborhood consistency constraints of the CRF-RNN optimization.

#### 4.3.2. Quantitative Analysis

Quantitative results for the overall performance and class-wise accuracy of material segmentation on the MINC dataset are given in Tables 3, 4, respectively. As Table 3 shows, FCN with CRF-RNN achieves 81.94, 74.19, 61.13, and 69.99% for the pixel accuracy, mean accuracy, mean IoU, and frequency weighed IoU, respectively, on the MINC dataset. Compared to FCN without CRF-RNN, FCN with CRF-RNN exhibits an improvement of 3.53, 2.28, 4.62, and 3.92%, respectively, for the pixel accuracy, mean accuracy, mean IoU, and frequency weighed IoU. As Table 4 shows, the class-wise accuracy for most classes is satisfactory, e.g., Hair (92.11%), Sky (96.71%), and Water (99.07%), but the performances for several classes are still inferior, especially Plastic (35.94%), due to the limited amount of training data. After introducing CRF-RNN following FCN-8s, the class-wise accuracy of each class increases by around 3–6%.

**Table 3:** The overall performance of material segmentation on the MINC dataset.

	**Pixel acc. (%)**	**Mean acc. (%)**	**Mean IU (%)**	**f.w. IU (%)**
FCN	78.41	71.91	56.51	66.07
FCN with CRF-RNN	**81.94**	**74.19**	**61.13**	**69.99**

*The best performance is in bold*.

**Table 4:** Comparison of the class-wise accuracy on the MINC dataset.

**Category**	**Brick**	**Carpet**	**Ceramic**	**Fabric**	**Foliage**	**Food**
FCN	61.02%	84.87%	72.95%	80.88%	**78.62%**	**65.04%**
FCN with CRF-RNN	**63.82%**	**86.18%**	**80.84%**	**84.26%**	77.38%	63.86%
**Category**	**Glass**	**Hair**	**Leather**	**Metal**	**Mirror**	**Other**
FCN	**67.28%**	92.08%	**72.05%**	72.35%	63.45%	39.44%
FCN with CRF-RNN	62.66%	**92.11%**	71.91%	**76.25%**	**69.81%**	**65.19%**
**Category**	**Painted**	**Paper**	**Plastic**	**P-Stone**	**Skin**	**Sky**
FCN	**90.62%**	56.83%	**43.94%**	51.75%	81.72%	95.96%
FCN with CRF-RNN	89.35%	**62.82%**	35.94%	**65.12%**	**83.37%**	**96.71%**
**Category**	**Stone**	**Tile**	**Wallpaper**	**Water**	**Wood**	**Mean**
FCN	62.68%	**66.16%**	**77.11%**	97.82%	79.28%	71.91%
FCN with CRF-RNN	**63.73%**	63.98%	69.17%	**99.07%**	**82.89%**	**74.19%**

*The best performance among the compared methods is in bold. P-Stone, Polished Stone*.

To the best of our knowledge, material segmentation is currently a less-studied research topic, and no good benchmark ranking has yet been deployed on the large-scale material datasets. The MINC dataset is the most popular material dataset for deep learning research, but it is a very coarse dataset, so a lot of data preprocessing and generation are required. The newly generated data in this paper are released as an important supplement to the MINC dataset, and the results provided can be employed as a baseline for future research. We hope that these can improve the benchmarking of research with respect to material classification and segmentation.

#### 4.3.3. Running-Time Analysis

We also provide the running-time performance of the proposed network in Table 5. The network is deployed using the 500 × 500 RGB images from the MINC dataset on a computer with an i7-6800k (3.4 Hz) 8-core CPU and NVIDIA TITAN X GPU (12 G). The running-time of FCN-based segmentation costs 0.13–0.15 s, and that of FCN with CRF-RNN costs 0.4s–0.6 s with 10 iterations or 0.2–0.3 s with five iterations. The running-time performance can be improved greatly if a smaller RGB image is used, which can enable real-time or near-real-time application of material segmentation.

**Table 5:** The running-time performance on the MINC dataset.

	**Running-time (s)**	**CRF iterations**	**Image size**
FCN	0.13–0.15	–	500 × 500
FCN with CRF-RNN	0.4–0.6	10	500 × 500
FCN with CRF-RNN	0.2–0.3	5	500 × 500

### 4.4. Material Reconstruction

As well as the evaluation on the MINC dataset, we further evaluate the proposed system in an industrial scenario, i.e., a real industrial room containing many different materials such as wood, brick, paper, metal, carpet, painted surfaces, and others. The system deploys a real-time 3D mapping of the room while simultaneously recognizing and labeling each point according to its material in the built 3D map. The network used in the system is only trained using the MINC dataset without fine-tuning on the real industrial data.

#### 4.4.1. Qualitative Analysis

**Figure 6:**
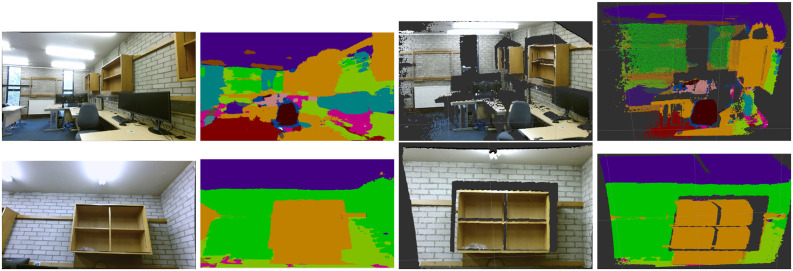
The qualitative results of each step generated by the material segmentation and reconstruction system. The first column are RGB images from Kinect2, the second column are material segmentation images, the third column are 3D point clouds, and the fourth column are 3D semantic point clouds.

**Figure 7:**
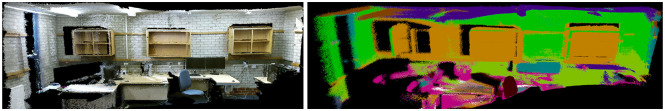
Material segmentation and reconstruction in an industrial scenario. **(Left)** Local 3D map. **(Right)** Local 3D semantic map.

**Figure 8:**
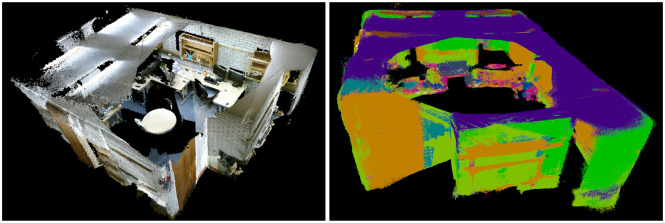
Material segmentation and reconstruction in an industrial scenario. **(Left)** Global 3D map. **(Right)** Global 3D semantic map.

We give the qualitative results of each step generated by the proposed system, i.e., original RGB image, material segmentation image, 3D point clouds, and 3D semantic point clouds in Figure 6. We also provide the local/global 3D map and local/global 3D semantic map of the industrial room in Figures 7, 8, respectively.

We can see that most of the materials are correctly classified and segmented in the dense 3D semantic map. However, some small objects are not labeled correctly due to there not being enough pixels provided in the original RGB image. The pixels at the border between two different materials are more easily assigned to the wrong labels. The domain variances, e.g., varying field of view, varying illumination, different imaging devices between the training and test data, also result in some wrong label predictions.

#### 4.4.2. Quantitative Analysis

We provide the quantitative results evaluated via pixel accuracy, mean accuracy, mean IoU, and frequency weighed IoU in Table 6. First, 40 key frames of 3D reconstruction in the industrial room were captured from RGB-D SLAM. Next, all the key frames were densely annotated according to the kind of material via JS Segment Annotator^4^. Finally, pixel-wise false or true numbers were counted between the corresponding pixels from predicted and ground-truth images.

**Table 6:** The overall performance of material semantic reconstruction in an industrial scenario.

	**Pixel acc. (%)**	**Mean acc. (%)**	**Mean IU (%)**	**f.w. IU (%)**
3D semantic reconstruction	80.10	58.75	39.45	68.76

As Table 6 shows, we achieve 80.10, 58.75, 39.45, and 68.76% for the pixel accuracy, mean accuracy, mean IoU, and frequency weighed IoU, respectively, tested in the industrial room. The pixel accuracy (80.10%) achieves a satisfying level, but the mean accuracy (58.75%) is much lower than the reported result for MINC evaluation (76.87%). Because we only tested 40 samples, there is a large variance in material detection rates. The pixel-wise recognition and segmentation accuracy of some materials, e.g., Paper (6.78%) and Mirror (0%) is very low. However, a mirror appears in only one instance, so failure to recognize just one instance of Mirror generates an accuracy of 0% for that category, which misleadingly skews the overall mean accuracy score toward a low value. In addition, the domain variances, e.g., varying field of view, varying illumination, and different imaging devices between the training and test data, also decrease the performance tested in the industrial room because the network is only trained using the MINC dataset without fine-tuning using the industrial data.

#### 4.4.3. Running-Time Analysis

The whole system is deployed on a computer with an i7-6800k(3.4 Hz) 8-core CPU and NVIDIA TITAN X GPU (12 G). The IAI Kinect2 package^5^ is adopted to interface with ROS and calibrate the RGB and depth cameras of Kinect2. The network is implemented based on the Caffe^6^ toolbox and accelerated by CUDA and CUDNN. The overall system is implemented through C++ and GPU programming within the ROS^7^ framework.

We provide the running-time performance of the whole system in Table 7. The system running-time performance is about 2 Hz (10 iterations) or 4 Hz (5 iterations) using the QHD RGB and depth images from Kinect2. The 540 × 960 RGB images are first reduced to 500 × 500 RGB images for material segmentation and are then recovered to 540 × 960 RGB images for semantic reconstruction. The running-time performance can be boosted to around 10 Hz when the QHD RGB images are decreased to 224 × 224 RGB images, using five CRF-RNN iterations for material segmentation.

**Table 7:** The running-time performance of the proposed system.

	**Running-time (Hz)**	**CRF iterations**	**Image size**
FCN with CRF-RNN	∽ 2	10	500 × 500
FCN with CRF-RNN	∽ 4	5	500 × 500
FCN with CRF-RNN	∽ 10	5	224 × 224

As mentioned (Hermans et al., 2014), there is no necessity to segment all the frames for the RGB-D SLAM because most of the frames are abandoned and only a few key frames (about 20%) are used for dense 3D mapping. In this case, a 5–10 Hz running-time performance can basically meet the requirement of a real-time material semantic reconstruction.

## 5. Conclusions

In this paper, we propose a novel transfer learning method to determine material categories from RGB images. Our approach is data-efficient, with maximization of the utility of a fundamental computer vision dataset and coarse annotated data. Consequently, our approach shows strong effectiveness in solving real-world problems, where large-scale training datasets are not available.

Moreover, the material understanding proposed by the neural network is integrated with 3D dense reconstruction, and incremental dense material labeling of a 3D scene is performed. The running-time performance of the whole system can be boosted to approximately 10 Hz to satisfy the requirement of real-time applications. We report qualitative, quantitative, and running-time evaluation analysis of the proposed approach using both the public material dataset and real-world industrial data to verify the resultant segmentation accuracy and running-time performance.

The newly generated high-quality dataset, including RGB image patches and fully pixel-wise annotated RGB images, is released as an important supplement for the MINC dataset. Our approach has a good alignment with industrial applications, especially nuclear robotics. As an essential part of the EU H2020 RoMaNs project, the proposed system has the potential to demonstrate its capability of guiding robots to navigate in industrial scenes and manipulate objects in a self-occluded heap.

## Data Availability Statement

The raw data supporting the conclusions of this article will be made available by the authors, without undue reservation, to any qualified researcher.

## Author Contributions

CZ proposed the main method and designed the experiments under the supervision of LS and RS.

## Conflict of Interest

The authors declare that the research was conducted in the absence of any commercial or financial relationships that could be construed as a potential conflict of interest.

## Footnotes

^2^https://www.image-net.org/

^3^https://www.robots.ox.ac.uk/~vgg/research/very_deep/

^4^http://kyamagu.github.io/js-segment-annotator/

^5^https://github.com/code-iai/iai_kinect2/

^6^http://caffe.berkeleyvision.org/

^7^https://www.ros.org
